# Promoting holistic wellbeing during the polycrisis: An essay on inner development through the martial art of Wing Chun

**DOI:** 10.1371/journal.pmen.0000433

**Published:** 2025-09-10

**Authors:** Ross Watson, Andrew H. Kemp

**Affiliations:** 1 Rehabilitation and Recovery Service, Swansea Bay University Health Board, Swansea, United Kingdom; 2 School of Psychology, Faculty of Medicine, Health and Life Science, Swansea University, Swansea, United Kingdom; PLOS: Public Library of Science, UNITED KINGDOM OF GREAT BRITAIN AND NORTHERN IRELAND

## Abstract

This essay explores the traditional martial art of Wing Chun through a psychological lens of inner development for holistic wellbeing. Originating in Southern China, Wing Chun emphasises fluidity, adaptability, and effectiveness, guided by five core principles: Simplicity, Practicality, Efficiency, Effectiveness, and Directness (SPEED). Grounded in Buddhist and Taoist philosophies, these principles provide a foundation for understanding and promoting psychological wellbeing. We propose a novel psychological framework that maps these principles to psychological constructs, including **R**adical non-attachment, **E**mbodied empowerment, skilful **A**daptation, self-**C**ontrol, and psychological **H**ardiness (REACH). Each construct within the REACH model functions synergistically, providing an opportunity for holistic cultivation of a resilient and adaptable mindset, grounded in mindful awareness, psychological flexibility, and fierce compassion. Radical nonattachment (underpinned by the principle of simplicity) facilitates a socially-engaged mindfulness, supported by a compassionate awareness of self, others and nature. Embodied empowerment (effectiveness) enables navigation of internal and external cues with greater agency while promoting a deeper sense of groundedness and engagement with the world. Skillful adaptation (practicality) promotes flexibility, creativity and the experience of psychological flow, supporting dynamic engagement with the environment. Self-control (efficiency) supports nonconscious regulation, facilitating purposeful goal-setting with reduced cognitive effort. Psychological hardiness (directness) supports the development of fierce compassion, transforming adversity into growth and enhancing capacity for working toward social justice and resisting dominant social narratives. Presented as an opportunity to promote power resources, these interrelated constructs bridge martial practice, mindful awareness, and social engagement, providing a robust foundation for psychological interventions supporting inner development and wellbeing at multiple scales, focused on self, others and nature.

“... *martial arts can become a vehicle and pathway to reducing suffering, promoting well-being and flourishing at a personal and social level*…”.

— Neil Clapton and Syd Hiskey, 2020

## Introduction

As the converging pressures of climate disruption, geopolitical instability, technological transformation, and global food system fragility give rise to what has been termed the polycrisis [1,2], there is a growing recognition that responses to psychological distress must extend beyond clinical symptom reduction. Contemporary frameworks such as the Broaden-and-Build Theory [3], Self-Determination Theory [4], and Seligman’s PERMA model [5] have emphasised the roles of positive emotion, autonomy, competence, relatedness and meaning in supporting flourishing. Yet even these perspectives, grounded in individual wellbeing, can fall short of addressing the psychological consequences of systemic, social, and existential challenges that now confront individuals and communities. In response, there is growing interest in integrative approaches that attend not only to psychological functioning but also to the cultivation of inner capacities for navigating complexity, sustaining meaning, and enabling purposeful action.

Traditional martial arts offer a compelling pathway. Rooted in ancient systems of self-cultivation, they combine physical discipline with ethical principles, mindful awareness, and embodied responsiveness [6–9]. Scholars are increasingly examining the martial arts through contemporary psychological models [10–13], which highlight the role of martial arts in fostering psychological skills and qualities including emotion regulation, social connection, meaning and autonomy. Among various traditions Wing Chun stands out as an instructive case. Developed by the Shaolin Buddhist nun Ng Mui, it was designed as a pragmatic and efficient system of self-defence [7,14]. The art is often symbolised by the interplay of snake and crane, a metaphor that reflects Taoist principles of yin and yang: the flexible, coiled energy of the snake complementing the grace and measured precision of the crane. Rather than abstract philosophy, these dynamics are enacted through embodied practice, balancing yielding with intent, and softness with structure [7, 14]. As a system pioneered by a woman and grounded in principles traditionally associated with the feminine, Wing Chun provides an opportunity to rebalance strength and receptivity in ways that support individual and collective wellbeing amid relational and ecological challenges characteristic of the unfolding polycrisis.

A growing body of research supports the relevance of martial arts for mental health and psychological wellbeing. A recent systematic review and meta-analysis found that martial arts interventions produced small to moderate improvements in both mental health (d = 0.620) and wellbeing (d = 0.346), despite notable heterogeneity [15]. Additional studies point to improvements in emotion regulation, stress tolerance, and psychological resilience. For example, Oh et al. [11] define ego-resilience as capacity to flexibly adapt to shifting demands while preserving emotional stability. Findings suggest that even brief martial arts programmes can reduce perceived stress and enhance positive affect. Although ego-resilience was not directly increased in this study [11], it formed part of a sequential pathway, alongside stress relief and positive emotions, linking physical activity to wellbeing, positioning martial arts as a valuable means of supporting psychological resources during times of disruption and change.

These empirical insights are complemented by long-term ethnographic accounts of martial arts practice. Jennings et al. [9], in a six-year study of Wing Chun, describe the emergence of a ‘Wing Chun habitus’, an embodied disposition through which practitioners internalise philosophical values, develop purpose, and experience belonging. Hackney’s [13] concept of ‘martial virtues’ further underscores the role of martial arts in cultivating moral character, with qualities such as temperance, courage, justice, and wisdom emerging through sustained practice. Together, these perspectives frame martial arts not merely as techniques for self-defence, but as ethical systems and “technologies of the self” [6,16], through which practitioners learn to regulate affect, embody values, and cultivate a resilient sense of self.

Such practices are particularly relevant to working through the polycrisis, where wellbeing depends not only on self-regulation or psychological flexibility, but also on the ability to embed personal growth within wider social and ecological systems. The Power Threat Meaning Framework (PTMF) [17] reflects this shift, reframing mental health in terms of contextual, relational, and meaning-making processes. Rather than asking “What is wrong with you?”, the PTMF focuses on what has happened, what meaning is made of it, and what strengths individuals might be able to draw on. In this context, martial arts training may serve not only to enhance self-efficacy and resilience, but also to support social identity, belonging and collective capacity, resources that will be essential for navigating and transforming systems in crisis [18,19].

To consolidate these insights, we introduce a framework to guide understanding of the psychological resources Wing Chun may help foster. Building on its core principles of Simplicity, Practicality, Efficiency, Effectiveness, and Directness (SPEED) [7,20], we propose the REACH model for inner development: Radical non-attachment, Embodied empowerment, skilful Adaptation, self-Control, and psychological Hardiness (Table 1). These constructs can be understood as inner ‘power’ resources that extend beyond individual symptom management, enabling practitioners to navigate complexity, resist disempowering narratives, and sustain meaning-making under pressure. Grounded in the embodied, ethical, and philosophical dimensions of Wing Chun, REACH offers a way of conceptualising how a traditional martial art that integrates mind, body, and spirit may make a distinctive and underexplored contribution to holistic wellbeing. In doing so, it aligns with the Power Threat Meaning Framework’s emphasis on contextual strength-based approaches and resonates with the emerging Inner Development Goals, equipping individuals to respond constructively to the wider social and ecological challenges of our time.

**Table 1:** Summary of the REACH model and its links to the five Wing Chun principles (SPEED).

Psychological construct	Description & relevance to wing chun	Wing Chun Principle	Martial context	Context and comment
Radical non-attachment	Mindful engagement with challenges, essential for maintaining emotional calm and navigating life’s challenges.	Simplicity	Elimination of unnecessary actions, focused on straightforward, natural and uncomplicated movement.	Here, we combine ‘radical’ with ‘non-attachment’, reflecting its role in martial contexts. Wing Chun fosters mindfulness-in-action, engagement and responsiveness. Rooted in Buddhist teachings on suffering and interdependence, non-attachment reduces suffering and promotes equanimity and compassion. Cultivates a socially-engaged mindfulness.
Embodied empowerment	Embodied self-awareness, rooted in bodily sensations, supporting the experience of empowerment.	Minimum use of brute strength (effectiveness)	Generating force through natural body structures while deflecting or redirecting an opponent’s force.	Emphasis on ‘embodiment’ and ‘empowerment’ highlights the role of the body in self-knowledge through monitoring of internal states. A non-reflexive and responsive approach to confrontation, reduces tension and improves effectiveness. Develops a rich connectedness to the self, others and the world.
Skillful Adaptation	Ability to flexibly adjust to competing demands, shift perspectives, and reallocate mental resources.	Practicality	Techniques that are readily applicable and adaptable to real-world situations.	Skillful adaptation aligns with concepts from psychological science (e.g., acceptance, psychological flexibility) and the Taoist philosophy of Yin/Yang. Flexibility and balance can be achieved through the cultivation and application of both Yin (e.g., soft) and Yang (e.g., hard) qualities.
Self-Control	Non-reflexive regulation of emotions, thoughts, and behaviours.	Economy of movement (efficiency)	Optimised movement minimising unnecessary motion to conserve energy and maximise effectiveness.	While control is initially consciously initiated, with practice, Wing Chun practitioners learn to respond without thought, supporting economy of movement (efficiency) through ‘skillful absorption’ (or ‘psychological flow’) or ‘effortless action’ (‘wu wei’). Such non-reflexive states contribute to positive and pleasurable experience. Relates to the development of character including the capacity for self-regulation, aligning with virtues like temperance.
Psychological Hardiness	Capacity to face challenges with commitment, control and adaptability, mirroring Wing Chun’s focus on purposeful and decisive action.	Directness	Swift and concise execution of movement that occurs over the shortest possible distance and time, simultaneously deploying attack and defence.	Here we link the principle of directness to the cultivation of psychological hardiness, which involves commitment, control and confidence in the face of adversity. Through concise and purposeful actions, practitioners develop self-efficacy, resilience and the courage to engage with challenges, embodying the qualities of fierce compassion.

### REACH for inner development

The Wing Chun principle of simplicity is explored through the lens of radical non-attachment, a Buddhist-inspired concept that has influenced modern psychological approaches, including compassion-focused [e.g., 21] and acceptance and commitment [e.g., 22] frameworks. We use the word ‘radical’ to emphasise non-attachment in a martial context, where it signifies a profound ‘letting go’ of thoughts, emotions, and physical reactions to achieve clarity and precision in action. ‘Non-attachment’ is defined as a ‘flexible, balanced way of relating to … experiences without clinging to or suppressing them’ helping to release the individual from unhelpful mental fixations [23]. Radical non-attachment addresses ‘dukkha’, the Buddhist notion of suffering arising from attachment to desires and aversions [24,25], supporting the experience of equanimity (a state of mental calmness), considered to be a foundational element for psychological wellbeing [26]. Recent research suggests that non-attachment might mediate the effect of mindfulness and self-compassion on wellbeing [27,28].

In Wing Chun, simplicity involves the elimination of unnecessary movement and responses, focusing only on what is essential to achieve the desired outcome. Non-attachment is developed through the practice of *Chi Sau* (or ‘sticking hands’), a form of sensitivity training that develops concentration, bodily awareness, and responsiveness to a partner’s movements [7]. Psychologically, this stands in contrast to habitual tendencies to grapple with challenging internal experiences or to cling to unpleasant thoughts and emotions, as demonstrated in this engaging presentation and conceptual illustration [29]. By contrast, the principle of simplicity as a martial principle reflects the psychological capacity to remain centred amid the instinctive drive to suppress or control unpleasant experiences, which research suggests can lead to increased distress [30]. By training to respond directly without extraneous movement, practitioners learn the ability to act without unnecessary mental deliberation.

Scholars refer to simplicity as a virtue that is linked to happiness [31], suggesting that the cultivation of simplicity in Wing Chun may offer psychological benefits beyond its martial applications. Simplicity and mindfulness have been referred to as a “co-evolving dialectic” that fosters clarity and insight [32]. Mindfulness reduces reactivity to negative thought patterns and emotions, mitigating distress and promoting wellbeing [33]. As Low [34] observes, “detaching ourselves and not getting caught with the great number of things in our mind, we slow down, pay attention to our breathing, move slowly and deeply, and we reach new understandings about ourselves and the world around us.” Meta-analysis shows that mindfulness-based programmes enhance compassion [35], and both mindfulness and compassion are qualities developed during martial arts training [12,13,36,37]. Moving beyond benefits to the individual, such qualities are now being promoted through the lens of ‘inner development’ in order to support progress on the sustainable development goals [38–40] (see also https://idg.tools/). Parallel lines of research on ‘voluntary simplicity’ also referred to as ‘minimalism’ (a low consumption lifestyle that has been associated with mindfulness, [41]) report higher levels of psychological need satisfaction, which in turn relate to higher levels of life satisfaction and sustainable wellbeing [42,43].

The cultivation of radical non-attachment in Wing Chun, reflected in the practice of responding rather than reacting, aligns closely with embodied empowerment, a psychological resource that foregrounds agency through bodily awareness. This is developed through the principle of effectiveness, which emphasises economy of motion and the redirection of force over brute strength [20]. Practitioners learn to engage not through confrontation, but through attunement: developing an instinctive sensitivity to others’ movements and intentions [44]. This capacity for ‘bodily listening’, described as “an openness to others” through proprioception [45], enabling practitioners to respond fluidly to dynamic situations through somatic cues rather than deliberate cognition. Such embodied presence links to the experience of engaged spirituality, where proprioceptive awareness becomes a basis for ethical responsiveness and transformation. Drawing on Foucault, Placido and Pedrini [45] suggest that this form of listening marks a turning point: where felt truth becomes “embedded” in the self and grounds a new ethos.

This embodied empowerment has practical implications, particularly for those recovering from trauma or resisting social control. Research with survivors of gender-based violence suggests martial arts can restore agency, confidence, and a felt sense of safety, with one participant stating it “literally makes me feel harder to kill” [46]. Beyond individual healing, martial arts can also challenge “hegemonic neoliberal ideas of how the body and mind should be treated and connected” [47], offering a counter-practice to forms of biopower that regulate behaviour through normative ideals. In this way, Wing Chun fosters psychological and physiological safety while nurturing resistance to systemic structures that undermine autonomy and dignity.

Embodied empowerment is developed through focused, diaphragmatic breathing that is introduced in the first of the Wing Chun hand forms, *Siu Nim Tao*. Basic hand movements are paired with controlled breathing techniques, which involves inhaling through the nose and exhaling from the diaphragm [14], activating the vagus nerve (the primary nerve of the parasympathetic nervous system). The vagus nerve serves as a bridge between mind and body and supports the regulation of essential functions and stress responses [48,49]. Enhanced vagal function is measured by increased heart rate variability (HRV), a psychophysiological marker of wellbeing that has been associated with emotional regulation, social connectedness, and psychological flexibility [50–53]. HRV has also been shown to support the capacity for “radically embodied compassion”, a construct that is a quality rooted in bodily awareness and emotional resilience and is particularly relevant to the martial arts [12,37,54].

Heightened bodily awareness fosters a deeper mind-body connection, supporting a “care of the self”, a practice that underpins individual as well as collective and ecological wellbeing [6]. Foucault purportedly broke away from his earlier writings on how social institutions produce ‘docile bodies’ in order to maintain the social order, and introduced the notion of self-care and ‘how individuals shape and transform themselves in order to reach a given state of … happiness and wellbeing… ’ [6 p.3]. Intriguingly, self-care can evolve into new social movements that critique and resist neoliberal governmentality and new forms of ‘bio-power’. Martial arts provides opportunities for the lifelong development of mind-body relationships, social connection and community building, and lay dispositions, practices, and conditions that can ‘sow the seeds of environmental awareness’ [6,8]. Supporting these ideas, the climate-informed psychotherapist, Staunton [55] has argued that losing an embodied relationship with ourselves and the world “contributes to our failure to grasp the reality of the interlocking crises we face,” underscoring the broader impact of mind-body practices like Wing Chun and opportunities for inner development through such practice.

Moving beyond a Foucauldian lens, the operation of power in our lives has been explored through the Power Threat Meaning Framework [PTMF, 56]. This alternative non-diagnostic conceptual system reframes distress by asking the question not what is ‘wrong with you’ but ‘what has happened to you’ (i.e., how does power operate in your life?), ‘how does this affect you’, ‘what sense did you make of this’ and ‘what did you have to do to survive’. According to this framework, power refers to the various ways in which external forces shape experiences, emotions and behaviours. For instance, ideological power refers to the ability of dominant narratives, belief systems and cultural values to shape how people understand their experiences and the world around them. An example of ideological power is the neoliberal ideology, which emphasises personal responsibility over collective wellbeing. Importantly, the PTMF also encourages asking the question ‘what are your strengths?’ and ‘what access to power resources do you have?’. Training in Wing Chun provides an opportunity to develop embodied empowerment, a power resource that supports new connections to self, others and the world.

Wing Chun’s approach to self-defence is also practical and emphasises realistic, adaptable responses using natural body mechanics and flexible techniques [7,14,20]. The ability to adapt in real time is a practical skill that is grounded in dynamic, interdependent decision-making processes [57]. Such adaptation is supported by psychological flexibility, which refers to the capacity to fully engage with the present moment, including any mental states it evokes (i.e., thoughts and emotions), enabling individuals to adapt their behaviour in line with one’s goals and values [58–60]. Therefore, psychological flexibility could also be considered a power resource, supporting skillful adaptation [61].

Wing Chun practitioners focus on the most direct and efficient path in both attack and defence known as the centreline plane [7]. When an action is blocked, instinctive responses like *fan sau* (‘returning hand’) seek alternative paths of least resistance [7,62]. Each action informs the next and involves constant adaptation to and negotiation with the opponent’s shifting behaviour and evolving dynamics [57]. Such practices enhance cognitive and psychological flexibility, as practitioners learn to navigate dynamic exchanges through mind-body connection, fostering self-awareness and non-attachment with thoughts and emotions [7,63–65]. Skilful adaptation mirrors the mental processes of balancing demands, reallocating resources, and shifting perspectives [52]. The martial arts also improve cognitive flexibility [65,66], linking martial practice to enhanced executive functions. Despite conceptual differences [67], both cognitive and psychological flexibility involve responsive behaviour to environmental changes and rapid attentional shifts [52,67], supporting continual adjustment to unfolding dynamics of specific situations.

This adaptiveness translates into a broader psychological resilience through what has been described as ‘somatic metaphorism’ [68], referring to the development of interpersonal skills that are then applied more generally to and positively affect, daily life [69]. The benefits of psychological flexibility for wellbeing are well-established [52,60]. During the COVID-19 pandemic, for example, psychological flexibility correlated with positive traits like wisdom, optimism, hope, peaceful disengagement, and self-efficacy [60]. Heart rate variability (HRV) underpins psychological flexibility and is a key mechanism for promoting individual wellbeing, while also supporting enhanced connections to self, others and nature [52,53,70,71].

Skillful adaptation is rooted in Yin/Yang philosophy, balancing the complementary qualities of softness (Yin) and hardness (Yang) [7,72]. Softness supports practitioners to draw power from flexible strength rather than brute force, enabling skillful adaptation in response to the demands of moment-to-moment experience [57]. This Yin/Yang integration aligns with Neff’s [73] integration of tender and fierce compassion in which tender compassion (Yin) reflects empathy and adaptability, while fierce compassion (Yang) involves assertiveness and boundary-setting. Wing Chun fosters such balance between acceptance and a readiness to act, supported by psychological flexibility [74]. Recent work highlights a key role for harmony and balance within and across people, ecosystems and time in the promotion of what has been described as ‘sustainable wellbeing’, ‘ecological wellbeing’ or ‘planetary wellbeing’, a multi-levelled and system- informed conceptualisation of wellbeing to which ‘people and societies can and should aspire’ [75–77]. This aspirational, higher-leveled conceptualization of wellbeing is consistent with a radically alternative worldview that aligns with the philosophical thinking of Taoism as well as the sciences of evolution, ecology, and environment [78].

We now explore the principle of economy of movement, examining how a focus on efficiency and precision builds self-control, which can further support wellbeing. In Wing Chun, economy of movement refers to minimisation of unnecessary motion to maximise functional impact and conserve energy [20]. Self-control and (the related construct of) self-regulation are personal strengths and resources that research shows are essential for self-discipline across different life contexts [79]. Self-regulation in childhood, for example, has been linked to positive life outcomes, including academic achievement, mental health, and overall wellbeing [80]. Conversely, a lack of self-control in difficult situations often reveals deeper psychological vulnerabilities, such as a propensity toward shame or emotional reactivity, which are linked to hostile responses [81]. Criticisms of martial arts have highlighted potential for antisocial behaviours, aggression, and violence [82]. Yet, a growing body of evidence now demonstrates that martial arts can reduce aggression and support character development. For instance, a meta-analysis of studies involving children and adolescents [83] found martial arts to be an effective intervention for reducing aggression, violence, and other externalising behaviours. Research with young people also highlights improvements in self-control, reductions in bullying, and the cultivation of *wu de*, which refers to moral character developed through disciplined practice [84]. These findings align with Hackney’s [13,36] concept of martial virtues, including temperance, courage, justice, and wisdom, positioning martial arts as a context for ethical growth as well as physical skill. Even in correctional settings, martial arts training has been linked to improved emotional regulation and reductions in antisocial behaviour [85].

In psychology, self-control typically refers to the conscious regulation of behaviours and emotions, often understood as a finite resource that can be depleted through overuse [86,87]. By contrast, Wing Chun develops a more refined and automatic form of control, developed through sensitivity practices such as *Chi Sau* [88]. Rather than relying on conscious decision-making, practitioners develop an ability to respond instinctively to changes in their environment. This kind of control aligns with what Prycker [89] refers to as “unself-conscious control”: an adaptive and instinctive responsiveness that is embodied and skilful, yet unfolds without the need for ongoing self-monitoring or reflective effort. It supports agency through a state of presence in which action flows from embodied attunement. Drawing on concepts such as psychological flow and the Taoist idea of *wu wei* (effortless action), this perspective challenges the notion that control must be effortful to be effective.

Wing Chun explicitly cultivates this non-reflexive form of self-control through structured practices such as *Chi Sau*. Practitioners learn to synchronise bodily actions and situational awareness, developing an instinctive responsiveness that conserves both mental and physical energy, enhancing adaptability, reducing muscular tension and promoting psychological flexibility. This form of self-control contributes to the cultivation of broader character strengths and ‘martial virtues’, including temperance, courage, benevolence, wisdom, and social justice [12,13,36]. Such virtues facilitate psychological wellbeing, underpin prosocial behaviours and contribute to societal harmony [90,91]. This disciplined mindset supports informed decision-making over impulsive responses, equipping practitioners to prioritise long-term goals and sustaining wellbeing over time [77,92,93].

This form of self-control aligns closely with the psychological construct of flow, an intrinsically rewarding state of deep, effortless immersion in an activity [94]. Prycker [89] points out the absence of narrative self-awareness within flow states, proposing that self-control can emerge without conscious deliberation or self-evaluation. This observation parallels the Zen-influenced concept of *mushin* or ‘no mind’, and Taoist principles of *wu wei* (effortless action) and *ziran* (natural spontaneity), all of which are echoed in the Wing Chun martial art [7,95,96]. *Chi Sau* practice exemplifies these principles through relaxed, efficient movements that redirect an opponent’s energy with minimal effort and without invoking conscious intent.

Psychological flow is considered to be essential for happiness and overall wellbeing [94,97]. It involves complete immersion and intrinsic motivation in an activity, fostering calmness and stability even in stressful situations. These benefits can extend beyond the training context, reflected in improved executive functions, emotional regulation, and general life satisfaction [65,98]. Activities fostering flow states have been associated with heightened environmental awareness and increased pro-environmental behaviours [8,43], reinforcing their potential for cultivating wellbeing not just at the personal level, but across social and ecological systems [99].

We now explore the principle of directness and its relationship with psychological hardiness, another quality that supports opportunities for inner development. Psychological hardiness is a component of personality that moderates the effects of stress on health. Originally comprised of three core dimensions, including commitment, control, and challenge, it was later expanded to include confidence [100–102]. Commitment reflects persistent engagement and dedication to goals, even amidst adversity, while challenge represents the capacity to reframe stressors as opportunities for growth. Control embodies both mastery over life circumstances and the effective regulation of emotions, nurturing a belief that outcomes depend on one’s own actions. Confidence encapsulates self-assurance in abilities and interpersonal effectiveness, empowering individuals to face new or challenging situations decisively. Collectively, these dimensions underpin psychological resilience, enabling individuals to proactively engage with stressors and transform them into growth opportunities [101,103,104], underpinning improvements in psychological wellbeing.

Directness in Wing Chun is exemplified through precise, intentional movements executed along the shortest and most efficient path between the practitioner and their opponent [7,20]. Central to Wing Chun’s effectiveness, directness integrates attack and defence into simultaneous, fluid actions [14,72]. For instance, when a practitioner simultaneously deflects an incoming strike and counters with their own strike, they exemplify directness, resolving conflict decisively to prevent prolonged confrontation and greater harm. Such directness is a form of ‘radically embodied compassion’ [12] or ‘fierce compassion’ [73], reflecting a commitment to protecting oneself (and others) with the least possible harm. Fierce compassion [73] is characterised by empowerment, bravery, and clarity, providing confidence to act assertively (empowerment), courage to face adversity directly (bravery), and the wisdom to guide decisive action (clarity). This combination of tender and fierce compassion harmonises with Yin/Yang philosophy and is physically represented through the Wing Chun stance (*Yee Gee Kim Yeung Ma*), where a strong, stable lower body supports dynamic movement, while the upper body remains relaxed and responsive [14].

Psychological hardiness shares similarities with the Finnish cultural concept of *Sisu*, which refers to a resolute determination and ability to draw strength from internal reserves when faced with adversity, emphasising perseverance despite significant psychological and physical challenges [105]. Hardiness reflects mental fortitude and embodied power, which will support psychological wellbeing as shown in published research. Individuals exhibiting higher levels of hardiness demonstrate reduced vulnerability to stress-related illness and emotional distress, perceiving and adapting to challenges more constructively [106,107]. Psychological hardiness also enhances resilience, emotional regulation, and overall quality of life [108,109]. Psychological hardiness offers a broader integrative framework than related constructs including grit and resilience. While grit focuses on perseverance towards long-term objectives despite setbacks [110] and resilience highlights equilibrium through positive adaptation to stress [111,112], psychological hardiness corresponds with mental toughness, which is enhanced through martial arts practice [101,113,114].

### Final reflections

In a world characterised by escalating challenges, including intergenerational inequalities [115], climate change [116], and ongoing humanitarian crises [117], traditional psychological and psychiatric interventions often fall short in addressing the complexity of wellbeing. This essay advocates for a more holistic approach, focused on the interconnectedness of how core Wing Chun principles [e.g., 7,20] may nurture psychological power resources. Moreover, we conceptualise Wing Chun’s transformative potential as a holistic practice integrating physical, mental, and spiritual dimensions that are aligned with emerging paradigms in mental health and wellbeing. Our framework reframes the traditional martial art through five intertwined capacities (Radical Nonattachment, Embodied Empowerment, skilful Adaptation, self-Control, and Hardiness, REACH), which together form a multifaceted and interconnected repertoire of psychological ‘power resources’. Synthesising Eastern philosophy and Western psychology positions the REACH model as a bridge between traditional wisdom and modern psychological science and lays a theoretical foundation for harnessing martial arts wisdom in the psychotherapy space or community wellness contexts.

Crucially, Wing Chun’s potential may extend beyond the individual by promoting relational and ecological wellbeing, through the cultivation of virtues such as compassion, wisdom and ethical engagement [12,13] and somatic metaphorism [68]. These qualities are essential in addressing the broader societal challenges, particularly the harmful impacts of power dynamics across personal, social, and ecological contexts [56,118,119]. In this way, our approach aligns with the Inner Development Goals initiative, which advocates for the promotion of inner capacities to achieve broader social and ecological change [38,39,120]. Wing Chun practice may therefore help individuals and communities mobilise psychological resources to address power imbalances, foster meaning in the face of adversity, and contribute to positive societal change.

Research is now needed to empirically evaluate the REACH framework and its impact across diverse populations and settings. In this reframed understanding, Wing Chun is not merely a martial art but a foundational repertoire of “power resources” for inner development, contributing meaningfully to issues relating to power relations, meaning in life, and holistic wellbeing. It offers a synergistic pathway for healing, growth, and flourishing across psychological, relational, and societal domains. When harnessed intentionally, a martial-based approach to inner development may offer a powerful catalyst for cultivating the inner capacities required to sustain collective resilience and foster just social transformation.
